# Application of tissue pneumoperitoneum technique around lymph nodes in thoracoscopic lung cancer resection

**DOI:** 10.3389/fonc.2024.1443088

**Published:** 2024-08-26

**Authors:** Fangqing Wang, Gang Chen, Weimin Ruan, Binkui Wang, Zhaowang Zhu, Weijian Hu, Sheng Chen, Lin Zang

**Affiliations:** Department of Cardiothoracic Surgery, The People’s Hospital of Tongling, Tongling, Anhui, China

**Keywords:** tissue pneumoperitoneum technique around lymph nodes, thoracoscopy, lung cancer, mediastinal lymph node dissection, lobectomy (Lob)

## Abstract

**Background:**

Thoracoscopic surgery is a primary treatment for lung cancer, with lobectomy and mediastinal lymph node dissection being the predominant surgical approaches for invasive lung cancer. While many thoracic surgeons can proficiently perform lobectomy, thorough and standardized lymph node dissection remains challenging. This study aimed to explore a safer and more efficient surgical method for mediastinal lymph node dissection in lung cancer.

**Methods:**

A prospective randomized controlled study was conducted, involving 100 patients with right lung cancer who were admitted to our hospital from January 2021 to April 2024 and met the inclusion criteria. These patients were randomly divided into an observation group (tissue pneumoperitoneum technique around lymph nodes group) and a control group (conventional surgery group). Thoracoscopic lobectomy and mediastinal lymph node dissection were performed. Intraoperative and postoperative related indicators were observed to validate the effectiveness and safety of the tissue pneumoperitoneum technique around lymph nodes.

**Results:**

The observation group showed a significantly shorter lymph node dissection surgery time compared to the control group, with a statistically significant difference (p < 0.05). The number of lymph nodes dissected in the observation group was significantly higher than that in the control group, with a statistically significant difference (p < 0.05). Although the observation group had slightly more mediastinal lymph node stations dissected than the control group, the difference was not statistically significant (p > 0.05). The total drainage volume within three days postoperatively was comparable between the two groups, with no statistically significant difference (p > 0.05). The observation group had shorter chest tube indwelling time and postoperative hospital stay than the control group, with statistically significant differences (p < 0.05). The incidence of surgical complications was similar between the two groups, and there were no perioperative deaths.

**Conclusion:**

The tissue pneumoperitoneum technique around lymph nodes is a more efficient method for mediastinal lymph node dissection in lung cancer, demonstrating safety and feasibility, and is worthy of promotion.

## 1 Introduction

Lung cancer ranks first in both incidence and mortality among malignant tumors (1, 2). A high incidence rate indicates that lung cancer is a common disease, while a high mortality rate reflects its significant harm and suboptimal current treatment outcomes. The pursuit of more effective treatments has become a hot topic in lung cancer research. Thoracoscopic anatomical lobectomy and hilar mediastinal lymph node dissection are the primary treatment methods for resectable lung cancer (3–5). Considering the risks associated with lymph node dissection, surgeons often resort to compromises such as lymph node sampling and non-systematic lymph node dissection (6–8). The main complications of mediastinal lymph node dissection include tracheal and bronchial injuries, recurrent laryngeal nerve injury, chylothorax, and hemorrhage (9). With the deepening of research on the lymph node metastasis patterns of lung cancer, surgeons have raised higher demands for mediastinal lymph node dissection. Theories such as skeletonized lymph node dissection, en bloc lymph node dissection, and non-grasping lymph node dissection are widely discussed (10–12),yet many surgeons find it challenging to meet these requirements. Our research team has pioneered the injection of medical carbon dioxide into the tissue surrounding mediastinal lymph nodes during thoracoscopic lymph node dissection for lung cancer. This method causes the fatty tissue around the lymph nodes to swell and expands the organ spaces, facilitating better exposure during lymph node dissection. This study aims to demonstrate the effectiveness and safety of this method, which has been approved by the hospital’s ethics committee(NO.2024015). China clinical trial registration number:MR-34-21-016006.

## 2 Materials and methods

### 2.1 General information

From January 2021 to April 2024, 100 patients with lung cancer underwent thoracoscopic lobectomy and hilar mediastinal lymph node dissection. Inclusion criteria were as follows: 1) age between 20 and 80 years, with no gender restriction; 2) preoperative or intraoperative pathological diagnosis of non-small cell lung cancer in the right bronchus; 3) clinical TNM staging of stage I or II; 4) ability to tolerate lobectomy. Exclusion criteria included: 1) severe cardiopulmonary dysfunction or other severe diseases that would likely preclude tolerance to thoracoscopic lobectomy; 2) tumors involving adjacent lobes requiring combined lobar resection; 3) patients unwilling to participate in the clinical study. Given the differences in lymph node dissection requirements between left and right lung cancers, this study exclusively selected patients with right lung cancer to ensure no significant differences between the two groups.

### 2.2 Sample size and case allocation

The required sample size was calculated using G*Power software, aiming for a high effect size of 0.8 between the treatment and control groups. With an alpha level of 0.01 and a power of 0.8, a minimum of 39 cases per group was determined. After ensuring the study outcomes and inclusion/exclusion criteria, we finalized 50 cases per group.

Patients were randomly assigned to either the observation group (mediastinal lymph node perisurgical tissue inflation method group) or the control group (conventional surgery group) using the envelope method. One hundred opaque envelopes and 100 sheets of paper were prepared, with 50 sheets labeled “mediastinal lymph node perisurgical tissue inflation method group” and the other 50 labeled “conventional surgery group.” These were randomly inserted into the envelopes, sealed, thoroughly shuffled, and numbered from 1 to 100. During each surgery, upon meeting the inclusion criteria, one envelope was opened sequentially, and the surgery was performed according to the group specified on the paper.

### 2.3 Surgical implementation

#### 2.3.1 Key surgical instruments

Thoracic Surgery Video-Assisted Thoracoscopic Surgery (VATS) System; Energy Devices include Hand-controlled Electric Hooks (L-shaped blade heads), Ultrasonic Knives, and Argon Knives; Conventional Instruments include Lymph Node Biopsy Forceps, Double-articulated Endoscopic Grasping Forceps and Dissecting Forceps, Metal VATS Suction Devices, Cutting and Suturing Instruments, and their corresponding Staplers.

#### 2.3.2 Observation group (perithymic pneumomediastinum surgical group)

**Figure 1:**
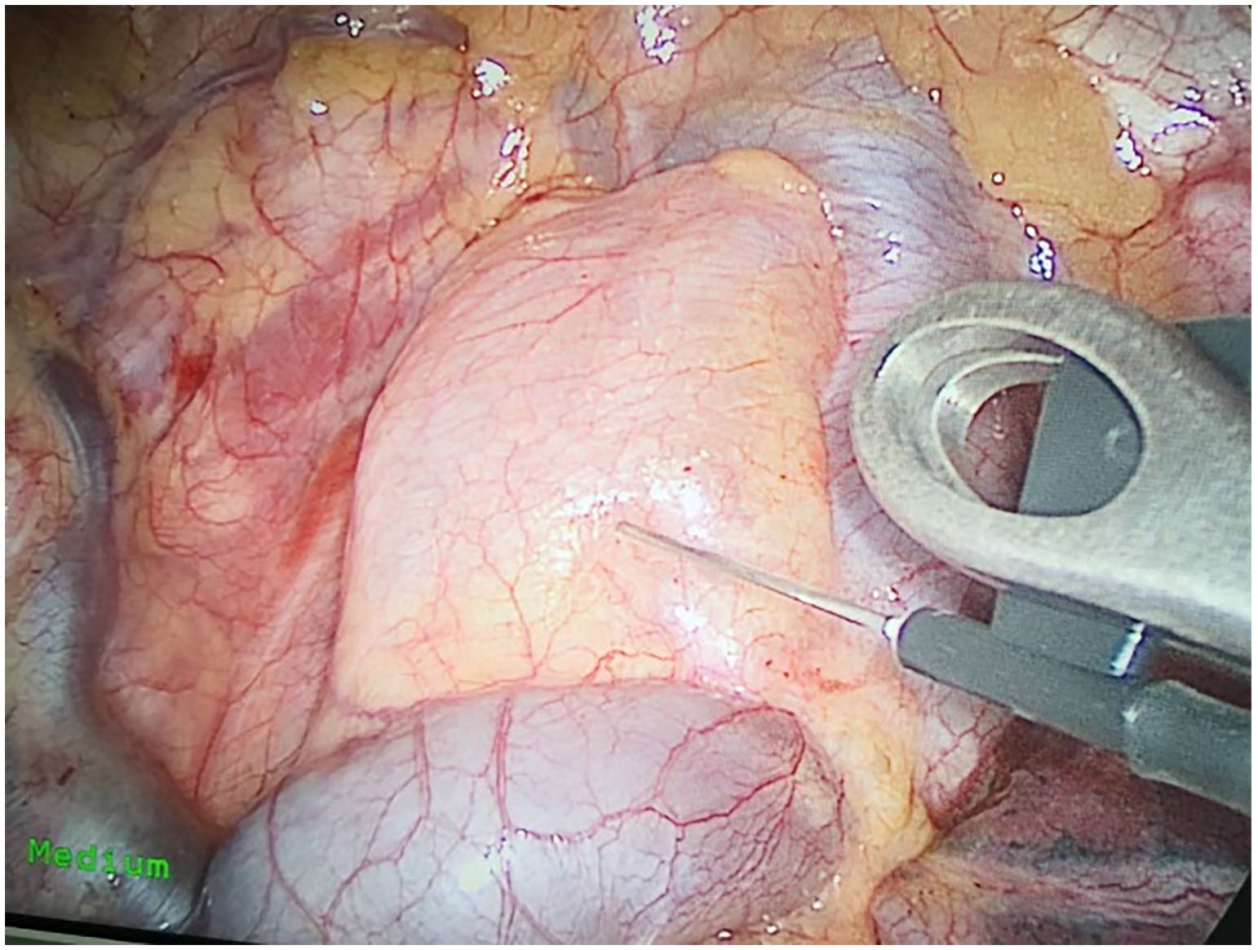
Injecting gas into the mediastinum.

**Figure 2:**
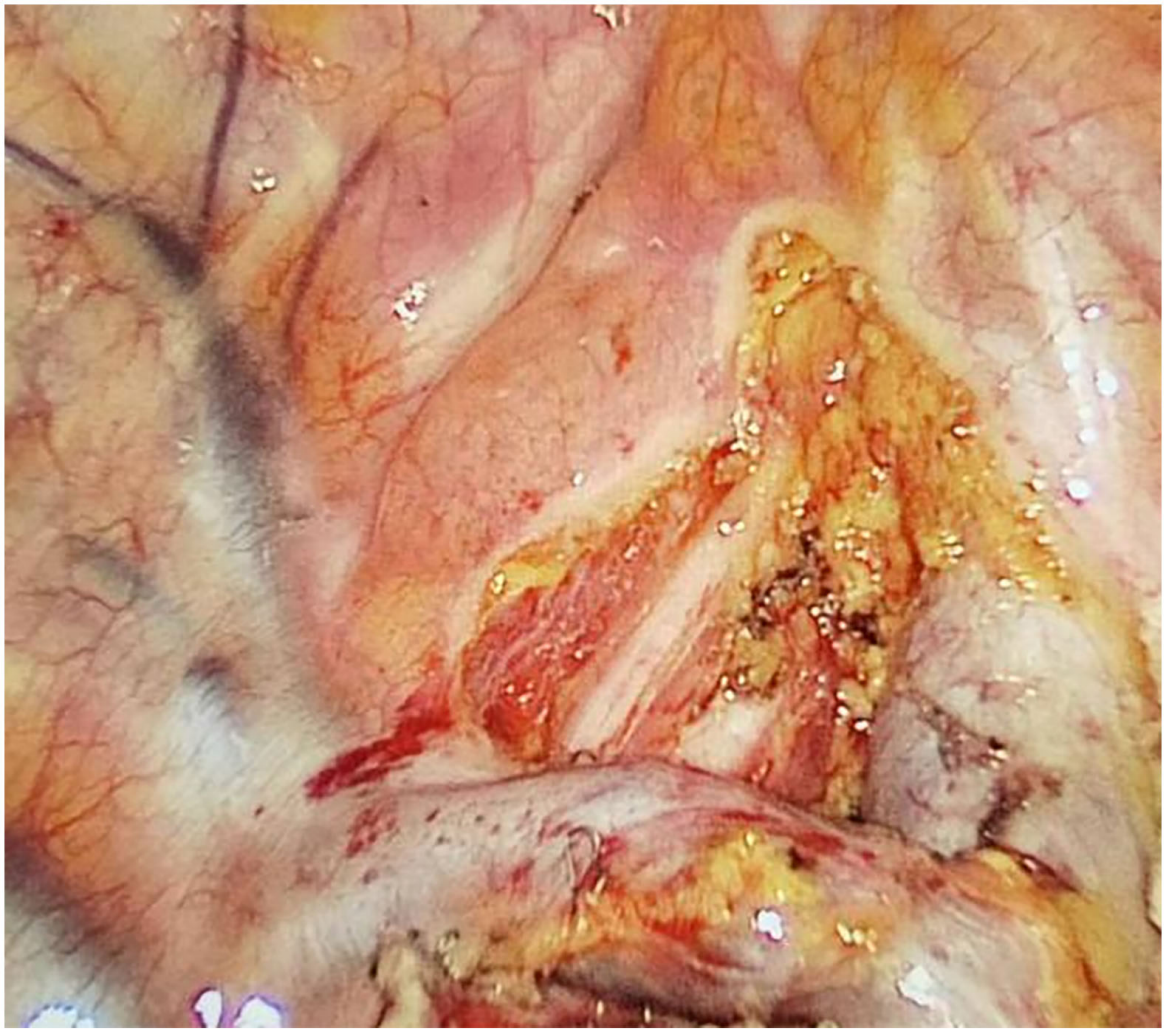
After lymph node dissection.

Preoperative or intraoperative pathological diagnosis of non-small cell lung cancer confirmed, followed by video-assisted thoracoscopic anatomical resection of a single right lung lobe. A 50ml syringe connected to a scalp vein needle was used as a self-made mediastinal gas injection device to infuse 50-100ml of medical carbon dioxide into the mediastinum (**Figure 1**). The puncture points were selected at the anterior trachea, posterior trachea, and carina, followed by systematic lymph node dissection. The procedure aimed for a non-grasping, en bloc, skeletonized lymph node dissection, including the right 2, 4, 7, 8, and 9 mediastinal lymph nodes (**Figure 2**).

#### 2.3.3 Control group (conventional surgical group)

Preoperative or intraoperative pathological diagnosis of non-small cell lung cancer confirmed, followed by video-assisted thoracoscopic anatomical resection of a single right lung lobe. Standard mediastinal lymph node dissection was performed, aiming for a non-grasping, en bloc, skeletonized lymph node dissection, including the right 2, 4, 7, 8, and 9 mediastinal lymph nodes.

### 2.4 Observational indicators

#### 2.4.1 Duration of mediastinal lymph node dissection

Recorded from the initiation of mediastinal gas infusion (in the observation group) or the incision of the mediastinal pleura (in the control group) to the completion of lymph node dissection with satisfactory hemostasis.

#### 2.4.2 Postoperative drainage volume and chest tube retention time

Recorded the volume of drainage in the chest bottle and the number of days the chest tube was retained within 3 days postoperatively.

#### 2.4.3 Criteria for drainage tube removal

The criteria for chest tube removal are as follows: 1)Less than 150ml of drainage in 24 hours; 2) No air leakage from the pleural cavity; 3)A follow-up chest X-ray showing good lung expansion.

#### 2.4.4 Efficacy of lymph node dissection

Evaluated by combining postoperative pathology reports to count the number of mediastinal lymph node stations and individual nodes.

#### 2.4.5 Postoperative recovery and incidence of severe complications

Recorded the number of postoperative hospitalization days, with major severe complications including recurrent laryngeal nerve injury, respiratory failure, bronchopleural fistula, secondary surgery due to active hemothorax bleeding, and chylothorax.

### 2.5 Data analysis

Data were processed using SPSS statistical software. Quantitative data were presented as means and analyzed using the t-test; categorical data were presented as frequencies/rates (%) and analyzed using the chi-square test. P < 0.05 was considered statistically significant.

## 3 Results

### 3.1 Baseline characteristics comparison

There were no statistically significant differences in gender, age, body mass index, lung lobe resected, and pathological type between the two groups (P > 0.05). See **Table 1**.

**Table 1:** Comparison of baseline characteristics between the two groups.

Variables	Observation Group(*n*=50)	Control Group(*n*=50)	*t*/*χ^2^ value*	*P value*
Sex [*n*(%)]			0.161	0.688
Male	28(56.00)	26(52.00)		
Female	22(44.00)	24(48.00)		
Age( $\bar{x}\pm s$ , years)	61.36 ± 8.51	62.98 ± 9.83	0.881	0.380
Body Mass Index (BMI)( $\bar{x}\pm s$ , kg/m^2^)	23.51 ± 1.80	23.25 ± 1.97	0.689	0.492
Lobectomy[*n*(%)]			0.740	0.690
Upper Lobectomy	25(50.00)	29(58.00)		
Middle Lobectomy	5(10.00)	5(10.00)		
Lower Lobectomy	20(40.00)	16(32.00)		
Histologic Type[*n*(%)]			0.156	0.925
Adenocarcinoma	39(78.00)	40(80.00)		
Squamous Cell Carcinoma	7(14.00)	7(14.00)		
Other Types	4(8.00)	3(6.00)		
Pathological T Staging				
pT1	28(56.00)	29(58.00)		
pT2	20(40.00)	17(34.00)	0.309	0.758
pT3	2(4.00)	4(8.00)		
Pathological N Staging				
pN0	42(84.00)	43(86.00)		
pN1	7(14.00)	1(2.00)	0.711	0.479
pN2	1(2.00)	6(12.00)		
N upstaging				
N0→N1	7(14.00)	1(2.00)		
N1→N2	1(2.00)	6(12.00)		

### 3.2 Comparison of surgical data between the two groups

Both groups successfully completed video-assisted thoracoscopic lobectomy and mediastinal lymph node dissection without conversion to open thoracotomy. The observation group had marginally better intraoperative blood loss than the control group, but the difference was not statistically significant (p > 0.05). The observation group had a shorter duration of mediastinal lymph node dissection, and a higher number of lymph nodes dissected compared to the control group, with statistically significant differences (p < 0.05). The observation group also had slightly more lymph node stations dissected than the control group, but the difference was not statistically significant (p > 0.05). See **Table 2**.

**Table 2:** Comparison of surgical data.

Variables	Observation Group(*n*=50)	Control Group(*n*=50)	*t value*	*P value*
Intraoperative Blood Loss (mL)	55.40 ± 40.05	64.50 ± 53.94	0.985	0.340
Duration of Mediastinal Lymph Node Dissection (min)	30.96 ± 9.90	40.78 ± 10.22	4.880	0.000
Number of Mediastinal Lymph Nodes Dissected	8.96 ± 3.63	7.26 ± 3.36	2.434	0.016
Number of Mediastinal Lymph Node Stations Dissected	4.06 ± 0.87	3.68 ± 1.06	1.964	0.052

### 3.3 Comparison of postoperative recovery between the two groups

The total drainage volume on postoperative day 3 was comparable between the two groups, with no statistically significant difference (p > 0.05). The duration of chest tube placement and postoperative hospital stay were both shorter in the observation group compared to the control group, with statistically significant differences (p < 0.05), as shown in **Table 3**. Each group had one case of recurrent laryngeal nerve injury. There were no cases of respiratory failure, bronchopleural fistula, secondary surgery due to active hemothorax, or chylothorax. There were no perioperative mortality cases.

**Table 3:** Comparison of postoperative recovery.

Variables	Observation Group(*n*=50)	Control Group(*n*=50)	*t value*	*P value*
Volume of Drainage on Postoperative Day 3 (mL)	690.00 ± 322.54	693.00 ± 347.58	0.043	0.966
Duration of Chest Tube Placement (d)	5.36 ± 3.49	7.76 ± 5.71	2.535	0.012
Postoperative Hospital Stay Duration (d)	6.80 ± 3.70	9.24 ± 6.06	2.429	0.017

## 4 Discussion

Thoracoscopic surgery is a primary treatment for lung cancer, with lobectomy and mediastinal lymph node dissection being the predominant surgical approaches for invasive lung cancer (3–5). Many thoracic surgeons can proficiently perform lobectomy, but comprehensive and standardized lymph node dissection remains challenging. In this study, the author referred to American and European guidelines and recommendations to perform systematic lymph node dissection. For right-sided lung cancer radical surgery, lymph nodes from stations 2, 4, 7, 8, and 9 on the right side were dissected, with a minimum of 6 lymph nodes removed to facilitate accurate staging assessment. Lymph nodes 3a and 3p were not dissected, as current international guidelines do not consider them mandatory for dissection (13–18).

Mediastinal lymph node dissection may increase surgical risks (9, 19), including: 1) prolonged operative time, elevating anesthesia risks; 2) vascular injury, leading to increased intraoperative blood loss; 3) injury to the recurrent laryngeal nerve causing hoarseness, injury to the vagus nerve cardiac plexus resulting in arrhythmias during and after surgery, and injury to the vagus nerve pulmonary plexus leading to postoperative difficulty in coughing and sputum expulsion; 4) damage to the main bronchi and trachea, potentially causing bronchopleural fistula and chronic cough postoperatively; 5) injury to the thoracic duct and lymphatic vessels, resulting in postoperative chylothorax, necessitating secondary surgeries in some cases; 6) increased surgical field, possibly leading to greater postoperative drainage and prolonged hospital stays.

Considering the aforementioned risks, higher demands are placed on the surgical team, including: 1) proficiency in mediastinal applied anatomy; 2) adept use of energy devices such as electrocautery hooks, ultrasonic scalpels, and argon beam coagulators; 3) extensive experience in thoracoscopic surgery. The potential risks and higher technical demands of lymph node dissection have limited its widespread adoption in primary hospitals. Therefore, the search for superior methods of lymph node dissection is particularly crucial.

Our surgical team experimented with injecting medical carbon dioxide into the mediastinum, observing a significant increase in the soft tissue spaces, which facilitated lymph node dissection. The primary advantages include: 1) Mediastinal tissue emphysema allows for lymph node dissection without clamping the nodes, making it easier to push or clamp surrounding tissues, enabling non-grasping lymph node dissection; 2) Accumulation of gas around the trachea, blood vessels, and nerves enhances their delineation, making anatomical layers clearer and reducing the likelihood of injuring critical structures, resulting in minimal bleeding and facilitating skeletalized lymph node dissection; 3) Tissue inflation around lymph nodes enlarges their volume, expanding the tissue spaces, making it less likely for lymph nodes to fragment and facilitating modular en bloc lymph node dissection. In our study, the observation group (surgical group using tissue inflation around mediastinal lymph nodes) showed a statistically significant reduction in lymph node dissection time compared to the control group (conventional surgical group), despite an additional minute for mediastinal inflation. In this study, the average time for mediastinal lymph node dissection was approximately 31 minutes, which is about 14 minutes shorter than that of the control group, representing a reduction of one-third. Reviewing relevant literature, the dissection time for right-sided mediastinal lymph nodes at comparable medical centers is approximately 30-60 minutes (3). With the increasing application of this technique and process optimization, the time for mediastinal lymph node dissection can be further reduced.The observation group also demonstrated superior lymph node dissection numbers, indicating more thorough dissection, likely due to clearer exposure of surrounding tissues. There was no significant difference in the number of lymph node stations between the two groups, as both underwent systematic dissection of lymph nodes 2, 4, 7, 8, and 9. Compared to other studies on mediastinal lymph node dissection techniques, this study demonstrates an advantage in the number of lymph nodes dissected, comparable to those of peer medical centers (20, 21).The observation group also outperformed the control group in terms of intraoperative blood loss, postoperative drainage days, and hospital stay, possibly due to clearer dissection views and more thorough dissection and ligation of bronchial arteries, lymph node-nourishing vessels, and lymphatic ducts. Fragmentation and residual lymph nodes often lead to increased bleeding and exudate, resulting in longer chest tube drainage and hospital stays, whereas en bloc removal of lymph nodes and surrounding tissues results in less bleeding. The average intraoperative blood loss in this study was approximately 55 ml, similar to the reported data from other comparable medical centers (22). Considering postoperative drainage efficacy and safety, this study adopted stricter criteria for chest tube removal. Other studies, based on their clinical experience and resource constraints, established more lenient removal standards, resulting in shorter durations of chest tube placement and hospital stay (23), However, under the same criteria for chest tube removal, our experimental group exhibited shorter durations of chest tube placement and hospital stay compared to the control group. The observed complications, including recurrent laryngeal nerve injury, respiratory failure, bronchopleural fistula, active thoracic bleeding, and chylothorax, were comparable between the groups, not only demonstrating the safety and feasibility of the method but also confirming its aforementioned advantages.

Upon reviewing the literature, our project team found no reports on the method of tissue inflation around mediastinal lymph nodes. The only related technique mentioned is the use of an inflation-assisted mediastinoscope for early esophageal cancer surgery (24, 25), which involves inflating the area around the esophagus to create a surgical pathway for esophagectomy. This method differs significantly from our lymph node dissection research, leaving us with no direct literature to reference. The areas requiring exploration include: 1) Selection of the optimal site for mediastinal gas injection; 2) Determining the appropriate gas type, specifically whether medical carbon dioxide is feasible; 3) Identifying the optimal volume of gas for the best effect; 4) Developing specialized surgical instruments for gas injection, beyond the use of scalpels, to enhance convenience and effectiveness.

**Figure 3:**
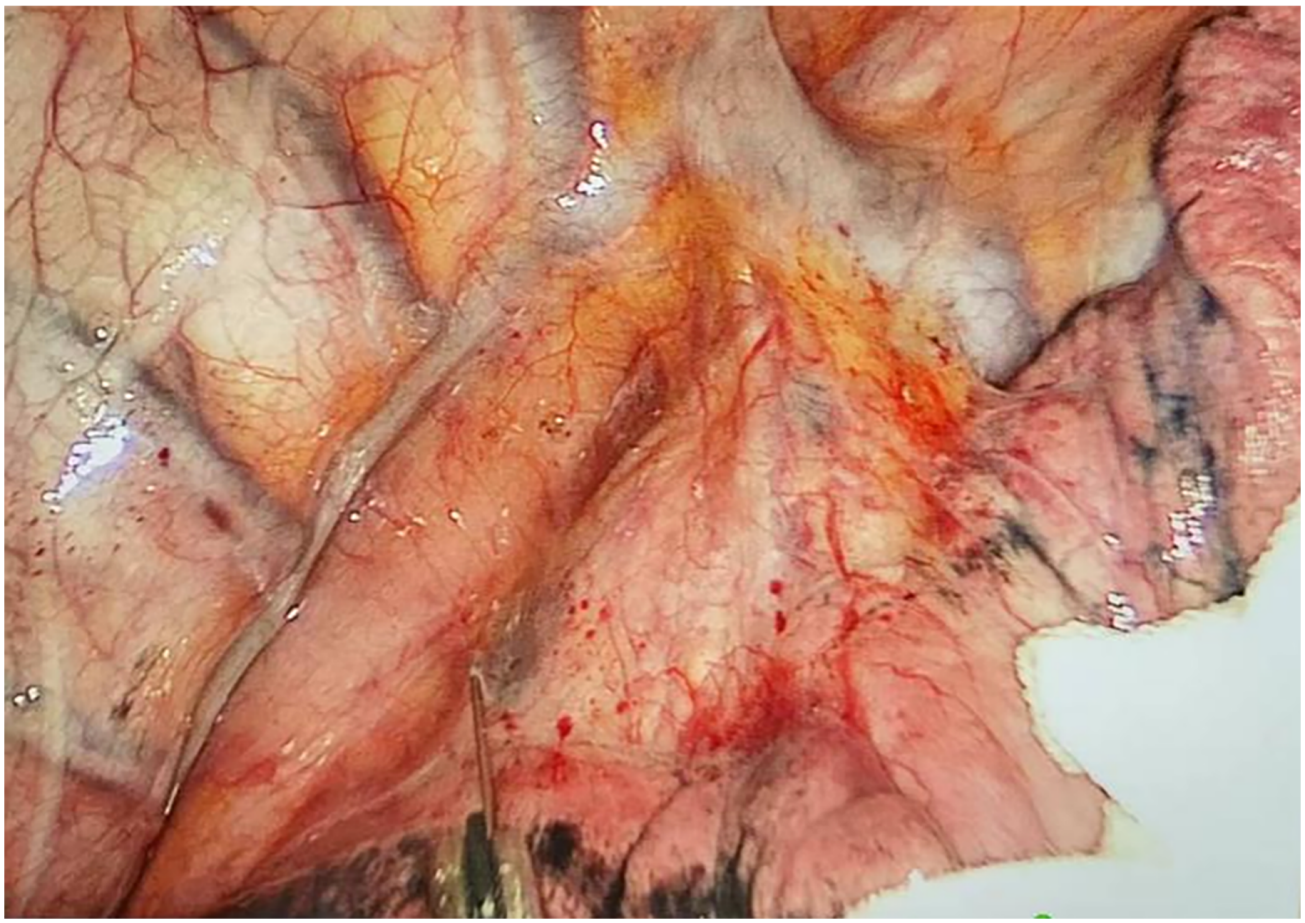
Before injecting gas into the mediastinum.

**Figure 4:**
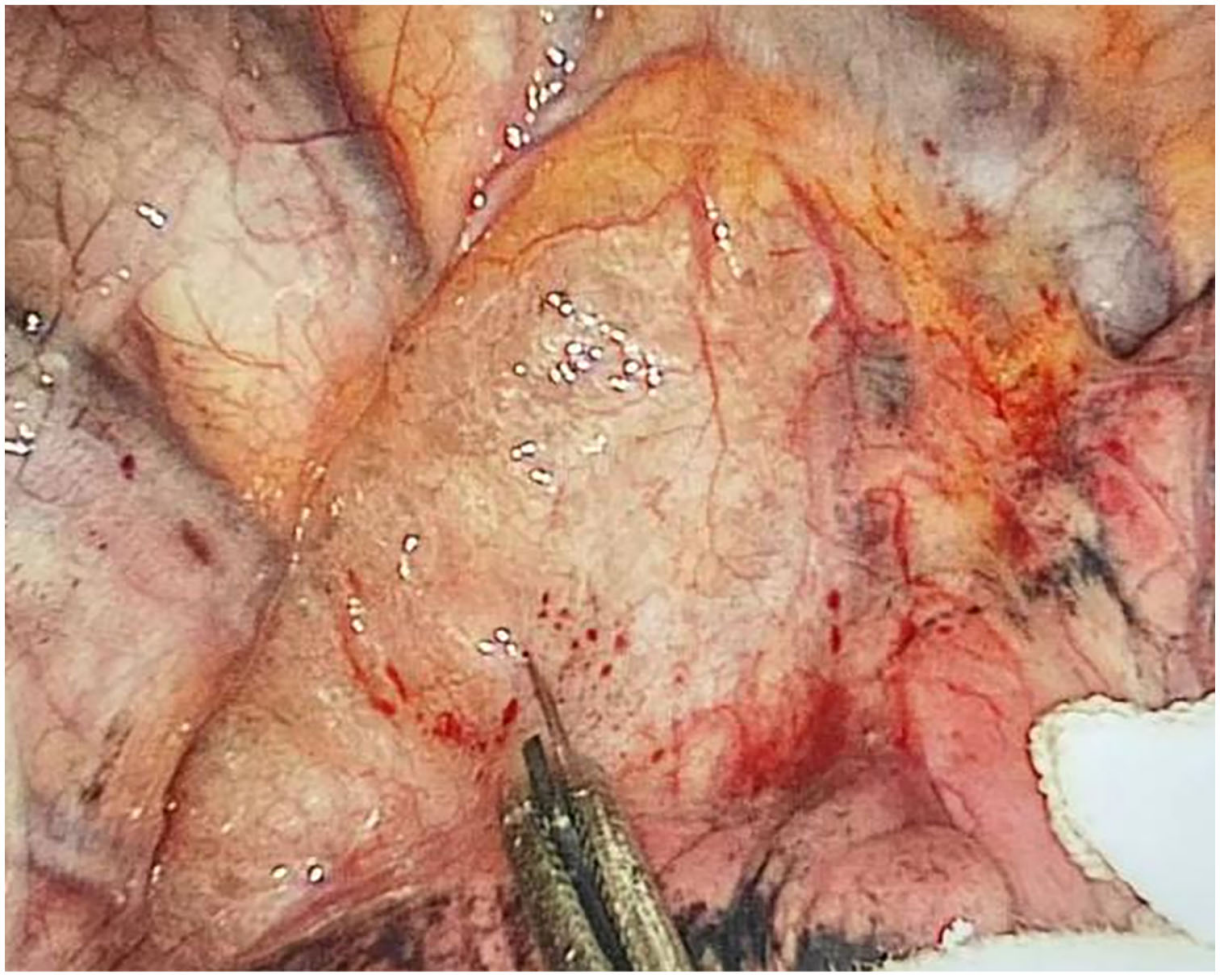
After injecting gas into the mediastinum.

**Figure 5:**
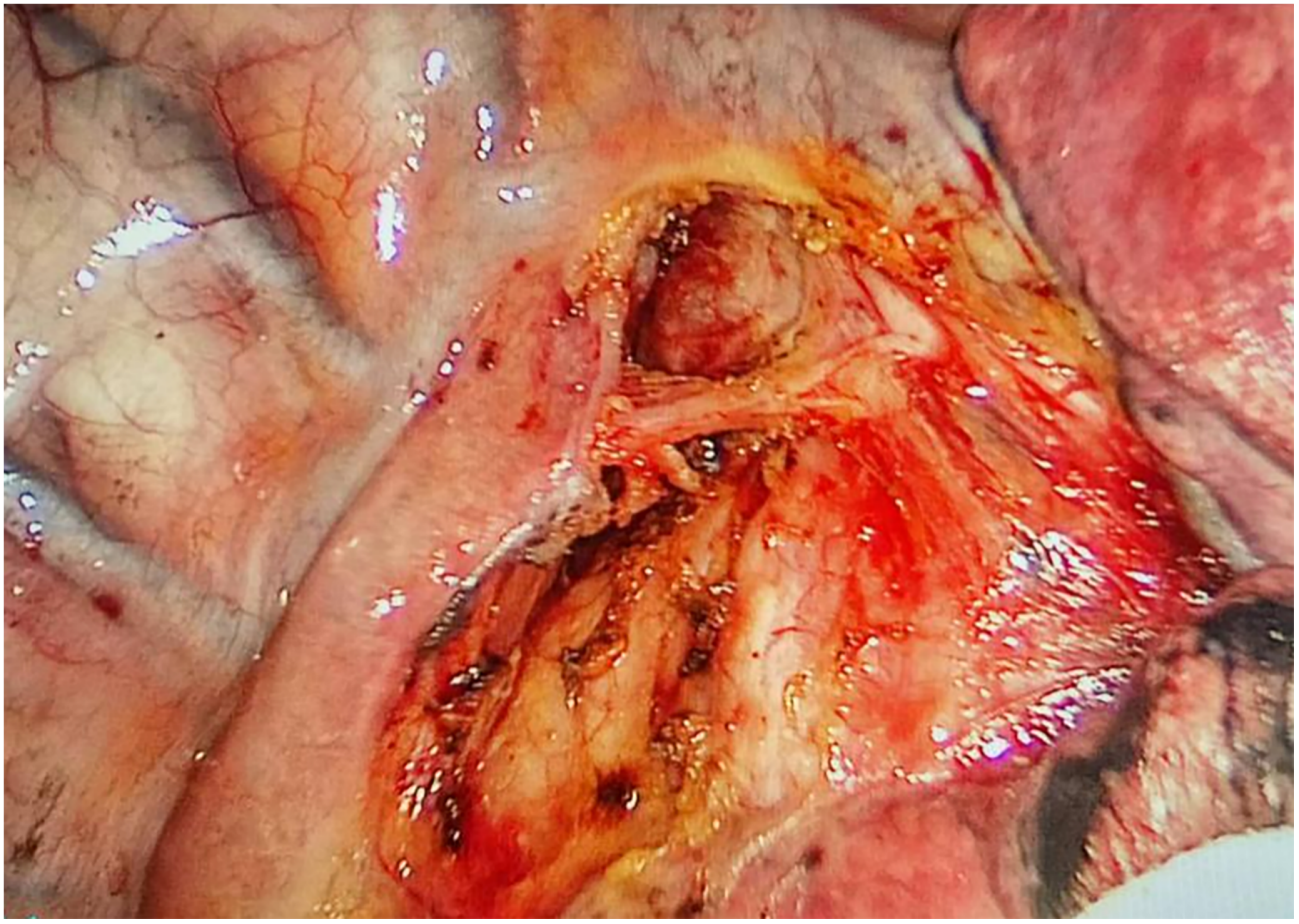
After lymph node dissection.

**Figure 6:**
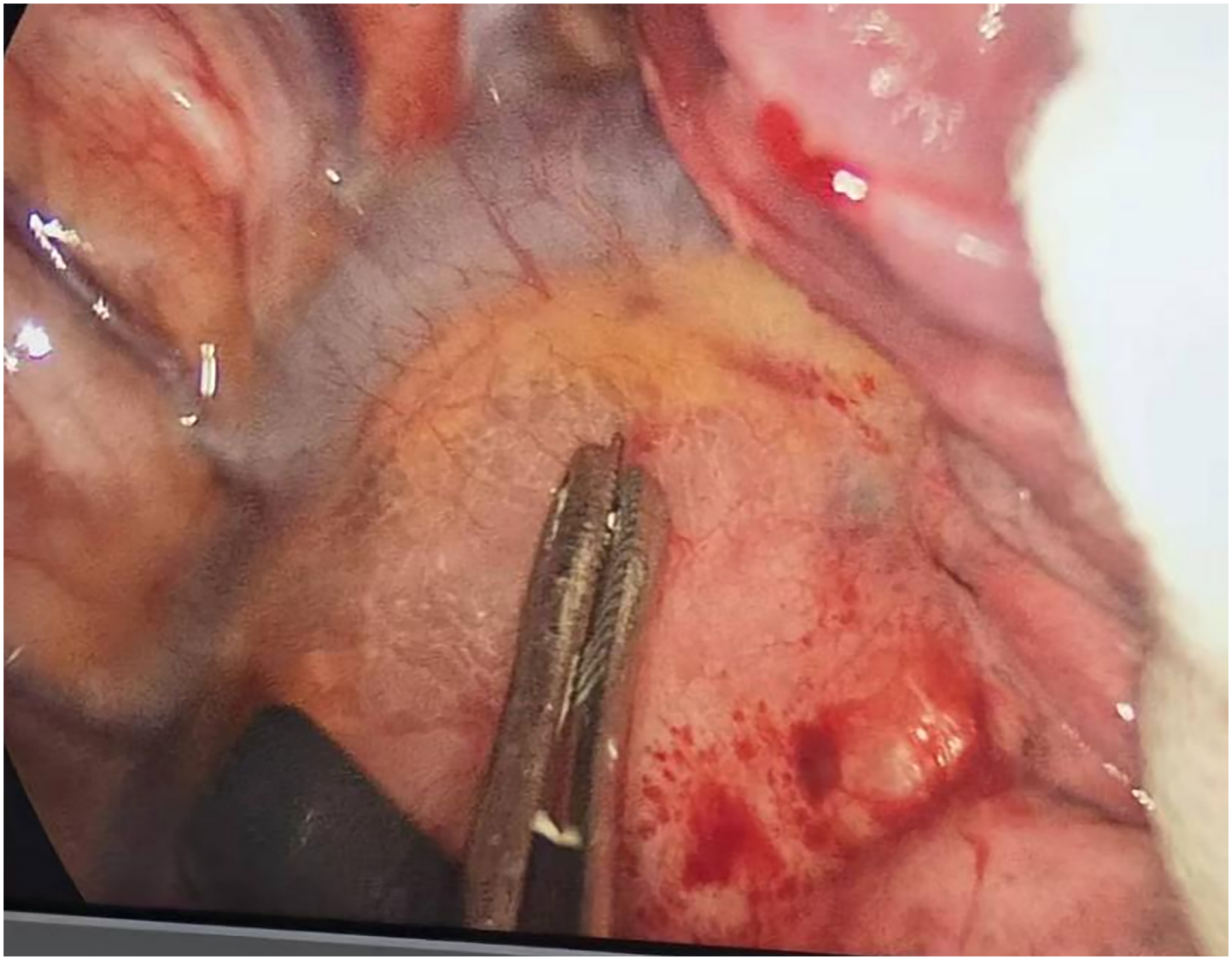
Injecting gas into the mediastinum.

Through our research, the selection of the gas injection site should adhere to the principles of safety and convenience. The following recommendations are made: 1) For lymph node groups 2 and 4 dissection, the injection site is recommended at the angle between the superior vena cava and the azygos vein, where the adipose tissue is thick, and the needle depth is easy to control (**Figures 1**, **2**). Alternatively, injection can be performed at the midpoint level of the superior vena cava, where inflation of the upper paratracheal space is more pronounced. The needle should always be directed towards the trachea to avoid accidental vascular penetration leading to bleeding, and accidental tracheal entry requires no intervention. 2) For lymph node groups 7, 8, and 9 dissection, the injection site should be below the carina, where the shadow of the lymph nodes is visible under the mediastinal pleura, facilitating easy gas injection (**Figures 3**–**5**). The needle should be directed towards the esophagus, and accidental esophageal entry requires no intervention. The puncture point can also be selected below the azygos vein, where the thick adipose tissue ensures safer needle insertion(**Figure 6**).

We have chosen medical carbon dioxide as the gas for injection due to its widespread application, easy availability, and its non-flammable nature, which allows for the safe use of energy devices such as electrocautery and argon beam coagulation without concerns (26). During the preliminary experimental phase, the project team initially experimented with injecting air, which yielded satisfactory surgical outcomes without adverse reactions. However, considering that air is not sterile and poses a risk of mediastinal infection, along with the fact that nitrogen in air is not readily absorbed by tissues, the team decided to opt for medical carbon dioxide. The team conducted trials with injections of 50ml, 100ml, 150ml, and 200ml of carbon dioxide, comparing the surgical outcomes. It was observed that injecting 100ml of carbon dioxide achieved the desired effect, with no significant advantages to injecting more gas. Conversely, injecting only 50ml sometimes resulted in suboptimal surgical results.

The project team utilized scalpels connected to syringes as the gas injection tools, employing thumb forceps or needle holders to grip the front end of the scalpels, exposing the needle tip by approximately 1cm, which should not be excessively long to prevent deep penetration into the mediastinum and potential injury to vital organs. The team has designed a specialized gas injection device and has applied for a patent (patent number: pending). Its effectiveness will be verified post-production experimentation, with ongoing efforts to refine and perfect the device’s details. The device must meet the following requirements: 1) It should have a long main body for ease of operation, aligning with the current needs of thoracoscopic surgery; 2) It must be capable of directly connecting to medical carbon dioxide gas supply systems, facilitating control over pressure and flow rate; 3) It should ensure safe puncture, avoiding accidental vascular or tracheal penetration.

This study represents a pioneering project with limited application experience and several shortcomings. The restriction to a small number of cases and the selection of radical resection of right pulmonary lobe cancer as the research subject may be inappropriate, as surgical trauma may differ among various pulmonary lobe resections, potentially affecting the comparison of data during and after lymph node dissection. Moreover, some scholars advocate for lobe-specific lymph node dissection or sampling for early-stage lung cancer. It is necessary for future research to delve into the radical resection of lung cancer in different lobes. Lymph node dissection during left lung cancer surgery also varies and requires well-designed studies for further validation. Currently, efforts are being made to apply this technique to mediastinal lymph node dissection in left-sided lung cancer resections. Due to anatomical constraints posed by the aortic arch, left recurrent laryngeal nerve (LRLN), and ductus arteriosus, when clearing lymph nodes 5 and 6, the inflation site is chosen at the intersection of the superior edge of the left upper pulmonary vein and the phrenic nerve, where the adipose tissue is thicker, making the puncture safer. For lymph nodes 7, 8, and 9 on the left side, the inflation site is selected at the superior edge of the left lower pulmonary vein, with the needle tip directed towards the esophagus, ensuring safe and convenient inflation. This method shows comparable efficacy on the left side as it does on the right, but further trials are needed to validate its effectiveness.

## 5 Conclusions

In summary, the gas inflation method around lymph nodes facilitates the realization of concepts such as skeletonized lymph node dissection, en bloc lymph node dissection, and non-grasping lymph node dissection. It is a safe and effective method for mediastinal lymph node dissection in lung cancer, meriting widespread application and promotion.

## Data Availability

The original contributions presented in the study are included in the article/supplementary material. Further inquiries can be directed to the corresponding author.
